# Comparative evaluation of visual outcomes and corneal asphericity after laser-assisted *in situ* keratomileusis with the six-dimension Amaris excimer laser system

**DOI:** 10.1371/journal.pone.0171851

**Published:** 2017-02-10

**Authors:** JunJie Piao, Ying-Jun Li, Woong-Joo Whang, Mihyun Choi, Min Ji Kang, Jee Hye Lee, Geunyoung Yoon, Choun-Ki Joo

**Affiliations:** 1 Catholic Institute for Visual Science, College of Medicine, The Catholic University of Korea, Seoul, Korea; 2 Department of Ophthalmology, Affiliated Hospital, Yanbian University Medical College, Jilin, China; 3 Department of Ophthalmology, Catholic Institute for Visual Science, Seoul St. Mary’s Hospital, College of Medicine, The Catholic University of Korea, Seoul, Korea; 4 Flaum Eye Institute, Center for Visual Science, The Institute of Optics, University of Rochester, Rochester, New York, United States of America; Weill Cornell Medical College in Qatar, QATAR

## Abstract

**Purpose:**

To compare the visual and refractive outcomes after laser-assisted in situ keratomileusis (LASIK) surgery for correction of myopia or myopic astigmatism using a six-dimensional Amaris excimer laser.

**Methods:**

In this retrospective cohort study, we enrolled 47 eyes of 28 patients (age: 19–36 years) with myopia or myopic astigmatism. We used the Custom Ablation Manager protocol and performed ablations with the SCHWIND AMARIS system. LASIK flaps were cut with an iFS Advanced Femtosecond Laser. Mean static (SCC) and dynamic cyclotorsion (DCC) were evaluated. Visual and refractive outcomes were evaluated during 6 months’ follow-up. Corneal asphericity (Q-value) was analyzed at 4 months postoperatively.

**Results:**

The spherical equivalent (SE) reduction was statistically significant reduce 1 day after refractive surgery (P < 0.001), with no additional significant changes during follow-up (P = 0.854). SCC registration rates were 81% in the Aberration-Free mode (AF) and 90% in the Corneal Wavefront mode (CW). SCC measurements were within ± 5 degrees in 57% (AF) and 68% (CW) of eyes. Mean DCC was within ± 1 degree in 96% (AF) or 95% (CW) of cases. At 6 months, the uncorrected distance visual acuity was 20/25 or better in all eyes. At last follow-up, both steep and flat keratometry values had significantly flattened in both groups (P < 0.001). Corneal asphericity also increased significantly during the postoperative period for an 8-mm corneal diameter (P < 0.001).

**Conclusions:**

LASIK for myopia or myopic compound astigmatism correction using the six-dimensional AMARIS 750S excimer laser is safe, effective, and predictable. Postoperative corneal asphericity can be analyzed by linear regression to predict the changes in postoperative corneal asphericity with this approach.

## Introduction

Given the increase in the number of myopic patients in the population, refractive surgery is the most commonly used surgical technique for correction of myopia or myopic astigmatism. Photorefractive keratectomy (PRK), laser in situ keratomileusis (LASIK), and laser epithelial keratomileusis (LASEK) are the most frequently used refractive surgery methods. Myopic-correction refractive surgery induces an increase in higher-order aberrations (HOAs), especially spherical aberrations, [1] which can degrade the visual quality with halos, glare, starbursts and night vision.

Thus, excimer laser refractive surgery has evolved from simple myopic ablations [2] to topography-guided [3] and wavefront-driven [4] procedures that use wavefront measurements of the whole eye [5] (Hartman-shack wavefront sensors) or customized ablation patterns based on corneal topography-derived wavefront analysis. [6,7]

The new generation of excimer laser platforms is designed to achieve a better ablation profile with a larger and optimized optical zone, with good tracking system to reduce the induction of aberrations and to obtain better postoperative visual acuity.

Among these, femtosecond laser-assisted LASIK has been improved by the development of femtosecond laser. Better control of flap creation with larger and more accurate femtosecond laser flaps may enhance performing myopia or myopic-compound astigmatism correction refractive surgery with a larger ablation zone (AZ) and thus may achieve more positive postoperative visual outcomes.

The aim of this retrospective study was to compare the visual outcomes and corneal sphericity after LASIK employing the Aberration-Free and Corneal Wavefront modes of the six-dimensional excimer laser treatment.

## Methods

### Patients and methods

This retrospective study included 47 eyes of 28 patients, from December 2012 to February 2016 at the Department of Ophthalmology, Catholic University, St. Mary’s Hospital, Seoul, South Korea. Informed consent was obtained from all the patients prior to the commencement of the study, and the study methods adhered to the tenets of the Declaration of Helsinki for use of human participants in biomedical research. The Institutional Review Board for Human Studies at Seoul St. Mary’s Hospital approved this study.

All surgery targeted emmetropia, and patients underwent a standard ophthalmologic examination preoperatively and at 1day, 1 week, and 1, 3, and 6 months after myopic correction refractive surgery. All eyes underwent uncorrected distance visual acuity (UDVA), best spectacle-corrected distance visual acuity (BSCDVA), manifest refraction (MR), cycloplegic refraction (CPR), slit-lamp examination of the anterior segment, intraocular pressure (IOP) measurement, and corneal asphericity was evaluated by Pentacam (OCULUS Wetzlar, Germany).

The study inclusion criteria were the requirement for myopic correction with refractive surgery, and normal preoperative topography. All patients had at least 1 year of stable refraction before undergoing refractive surgery. Exclusion criteria included ocular pathology, retinal disorders, ocular operation history, or common medical histories, such as diabetes, autoimmune pathologies, endocrine pathologies, dry eye symptoms, and insufficient follow-up. We also excluded patients with corneal instability, corneal haze, or other complications, as well as retreatment cases. Patients had to discontinue use of soft contact lenses (SCL) for at least 2 weeks and rigid gas permeable (RGP) lenses for at least 4 weeks prior to surgery.

### Surgical technique

All the treatment plan followed the Custom Ablation Manager protocol and ablations were performed using the AMARIS 750S excimer laser (SCHWIND Eye-Tech-Solutions, Kleinostheim, Germany). Aberration-Free mode involves ablation with optimized aspheric profile centered on the pupil center, while Corneal Wavefront mode involves ablation with corneal topography centered on the corneal vertex as measured by videokeratoscopy (Keratron Scout topographer, Optikon 2000 SpA) under photopic conditions (1500 lux), similar to the conditions under the operating microscope.^9^ All surgeries were performed by a single experienced surgeon (CKJ). Topical eye drops of proparacaine (Alcaine; Alcon-Couvreur, Puur, Belgium) was instilled as anesthetic. LASIK flaps were cut using the iFS Advanced Femtosecond Laser (Abbott Medical Optics, Inc., Irvine, CA, USA) with superior hinges, 100-μm flap thickness, and 8.4-mm or 8.5-mm flap diameters. After lifting the flap, ablation was performed with a 6.5-mm optical zone. The planned refractive correction (6.7–9.0 mm) of the ablation zone (AZ) was provided automatically with a variable transition zone (TZ) size.

### Statistical analysis

Data were recorded into an Excel spreadsheet database (Microsoft, Redmond, Washington, USA) and statistical analysis was performed using SPSS for Windows, version 18.0 (SPSS, Inc., Chicago IL, USA). The Shapiro-Wilk test was used to confirm normality of the data. Preoperative and postoperative data, or data from postoperative and follow-up visits, were analyzed using Student’s *t*-test. The Wilcoxon rank sum test was used for nonparametric analyses. Unpaired *t*-tests or nonparametric Mann—Whitney tests were used for comparing preoperative and postoperative parameters between the two groups. P-values of less than 0.05 were considered statistically significant.

## Results

This retrospective study included a total of 47 eyes, that underwent laser refractive surgery between December, 27, 2013, and January, 20, 2016. The demographic details, and the visual and refractive outcomes of the two groups are listed in Table 1. The two groups did not show any significant difference in any metrics (all P > 0.05, Table 1).

**Table 1:** Patient population and preoperative postoperative average values.

Parameter	Aberration-Free	Corneal Wavefront
No. eyes	26	21
Age (y)	25.92 ±5.67	26.90 ± 5.37
Gender (M/F)	9/17	5/16
Preoperative SE (range), D	-6.93 ± 2.73 (-1.88 to -12.00)	-6.90 ± 2.58 (-2.13 to -10.75)
Preoperative Cylinder (range), D	-0.82 ± 0.66 (0.00 to -2.25)	-1.25 ± 0.63 (-0.25 to -2.75)
Postoperative SE (range), D	-0.49 ± 0.59 (-1.88 to 0.25)	-0.11 ± 0.74 (-1.38 to 0.75)
Postoperative Cylinder (range), D	-0.63 ± 0.35 (0.00 to -1.50)	-0.65 ± 0.32 (-0.25 to -1.50)
Residual bed thickness (μm)	353.81 ± 43.69 (294.00 to 475.00)	350.19 ± 44.86 (226.00 to 455.00)
Pupil off-set distance, mm	0.21 ± 0.09 (0.02 to 0.36)	0.21 ± 0.11 (0.06 to 0.40)

F = female; M = male; SE = spherical equivalent.

The mean preoperative and postoperative steep and flat keratometry in both groups are presented in Table 2. At last follow-up, both steep and flat keratometry values had significantly flattened in both groups (P < 0.001). There was statistically significant flattened in the postoperative steepest keratometry reading (K_2_) in the Corneal Wavefront groups than the Aberration-Free groups (P = 0.035; data not shown).

**Table 2:** Mean preoperative and postoperative keratometry.

Parameter	Preoperative	Postoperative	*P*
Aberration-Free			
K_1_ (D)	42.64 ± 1.15 (40.50 to 45.75)	37.33 ± 2.59 (33.75 to 41.25)	< 0.001
K_2_ (D)	43.86 ± 1.40 (41.50 to 34.50)	38.09 ± 2.79 (34.50 to 42.50)	< 0.001
Corneal Wavefront			
K_1_ (D)	42.49 ± 1.27 (40.50 to 44.50)	37.16 ± 2.17 (34.00 to 41.50)	< 0.001
K_2_ (D)	44.04 ± 1.45 (41.50 to 45.75)	37.90 ± 2.22 (35.25 to 42.50)	< 0.001

K_1_ = flattest keratometry reading; K_2_ = steepest keratometry reading.

### Static cyclotorsion and dynamic cyclotorsion

**Fig 1:**
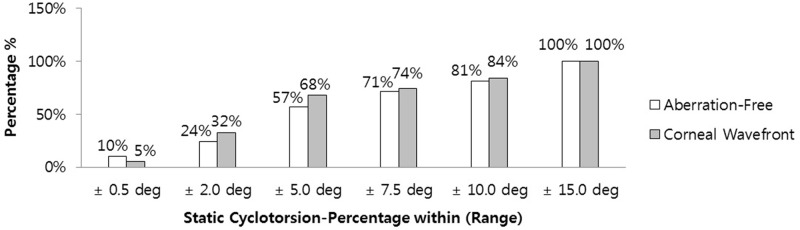
Distribution of the measured static cyclotorsion (SCC) from upright to supine position.

The successful registration rate for static cyclotorsion (SCC) was 81% (Aberration-Free) and 90% (Corneal Wavefront). A total of 57% of SCC measurements were within ± 5 degrees, two eyes (Aberration-Free) showed SCC values > 10 degrees, and three eyes (Corneal Wavefront) showed SCC values > 10 degrees (Fig 1).

**Fig 2:**
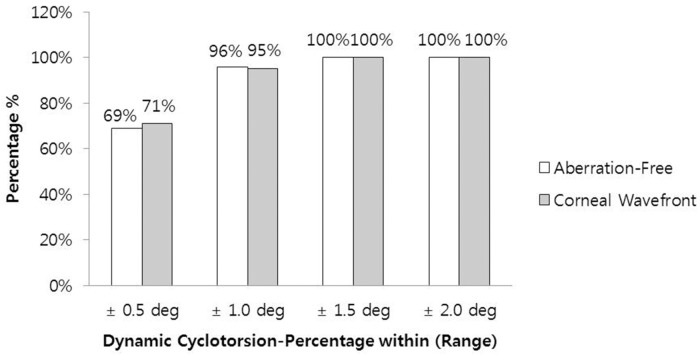
Distribution of the measured average dynamic cyclotorsion (DCC) during the ablation procedures.

The successful registration rate for dynamic cyclotorsion (DCC) was 100% in both groups. The mean DCC value during the treatment was -0.13 ± 0.51 degrees (range: -9.0 to +0.46 degrees) in Group 1, and -0.01 ± 0.55 degrees (range: -0.96 to +1.33 degrees) in Group 2. There was no significant difference between the two groups (P = 0.967). A total of 69% eyes (Aberration-Free) and 71% eyes (Corneal Wavefront) were within ± 0.5 degrees, and 96% eyes (Aberration-Free) and 95% eyes (Corneal Wavefront) were within ± 1.0 degrees (Fig 2).

### Visual acuity and refractive outcomes

**Fig 3:**
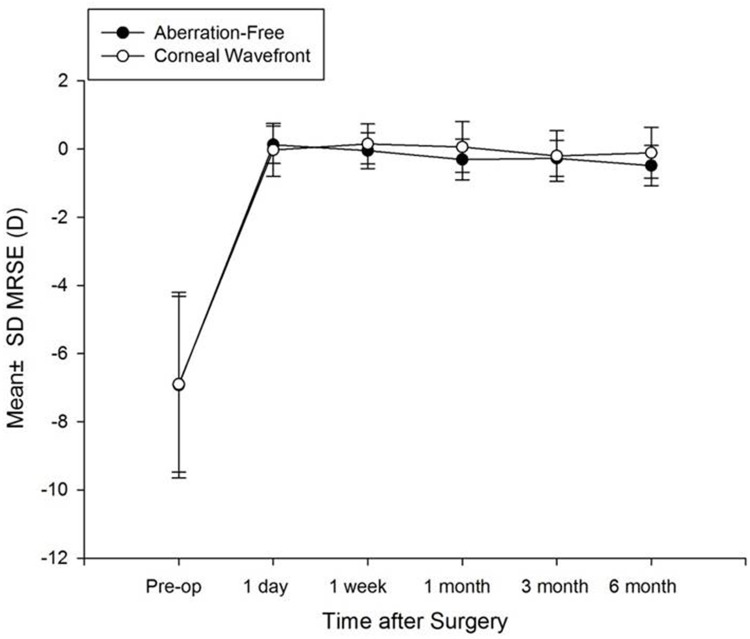
Stability of mean refractive spherical equivalent (MRSE) between preoperative and various postoperative and various postoperative visits between the two groups.

**Fig 4:**
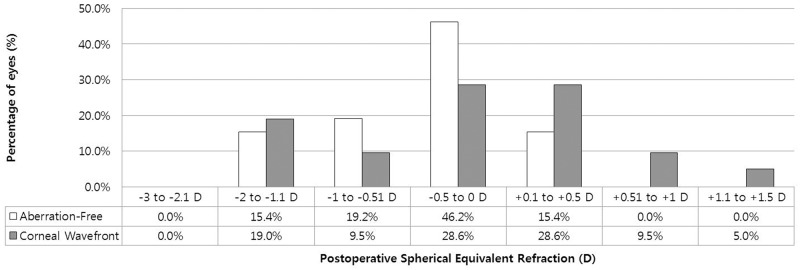
Distribution of the postoperative spherical equivalent (predictability) in the sample of myopia or myopic compound astigmatism eye undergoing laser-assisted in situ keratomileusis with six-dimensional Amaris laser platform.

**Fig 5:**
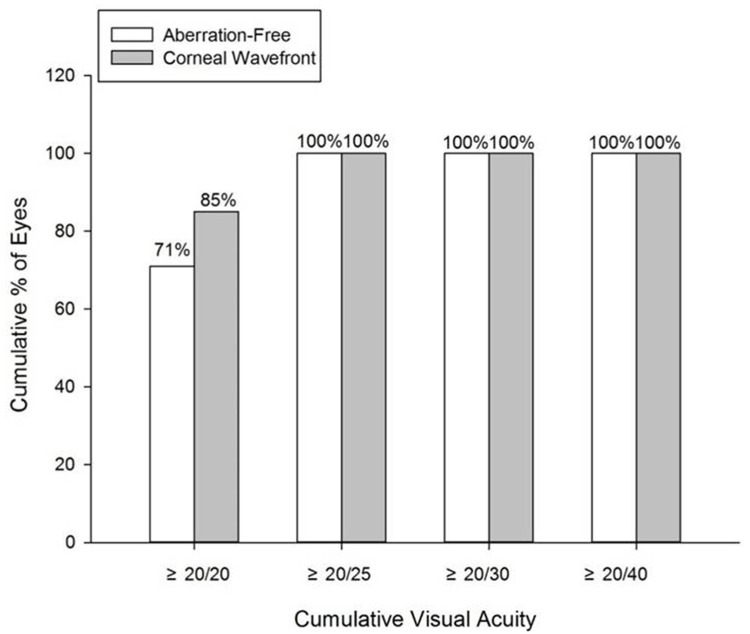
Distribution of uncorrected distance visual acuity (UDVA) (20/20 or better) between the two groups at 6 months postoperatively.

Fig 3 shows the spherical equivalent preoperatively, and postoperatively at 1 day, 1 week, and 1-, 3-, 6-month’s follow-up visits after refractive surgery in two groups. The spherical equivalent (SE) reduction was statistically significant at 1 day after refractive surgery (P < 0.001), with no additional significant changes during the remaining follow-up period (P = 0.854; Fig 1). A total of 15.4% of eyes (Aberration-Free) and 19% of eyes (Corneal Wavefront) were within -2 D to -1.1 D; 19.2% of eyes (Aberration-Free) and 9.5% of eyes (Corneal Wavefront) were within -1 D to -0.51 D; 46.2% of eyes (Aberration-Free) and 28.6% of eyes (Corneal Wavefront) were within -0.5 D to 0 D; 15.4% of eyes (Aberration-Free) and 28.6% of eyes (Corneal Wavefront) were within +0.1 D to +0.5 D. In the Corneal Wavefront group, 9.5% of eyes were within +0.51 D to +1 D, and 5% of eyes were within +1.1 D to +1.5 D (Fig 4). Fig 5 shows the distribution of UDVA at 6 months in the two groups. At 6 months, the UDVA was 20/25 or better in 100% of the eyes in both groups and 20/20 or better in 77% of the eyes in the Aberration-Free and 95% of the eyes in the Corneal Wavefront groups.

### Corneal asphericity

Corneal asphericity was analyzed by linear regression, and the 8-mm corneal diameter at 4 months was negatively correlated with the preoperative sphere and ablation zone (AZ). This relationship could be described by the following formula:

$${\text{Q}}^{\text{8mm}}{}_{\text{4m}}=\;3.19\;-\;0.301\;\times {\text{ SE}}_{\text{PRE}}-\;0.494\;\times \text{ AZ }\left(\text{Aberration}-\text{Free}\right)$$

The level of corneal asphericity was negatively correlated with the highest myopic refractive error (negative spherical equivalent) after LASIK. Specifically, we found that corneal asphericity increased by 0.301 with each diopter of SE correction in the postoperative period. However, postoperatively, corneal asphericity reduced by 0.494 with each increase of 0.1 mm in the AZ (Table 3).

**Table 3:** Linear regression analysis showing changes in asphericity for 8-mm corneal diameter (Aberration-Free).

SE (D)	AZ (mm)					
	7	7.2	7.4	7.6	7.8	8
-9.00	2.44	2.34	2.24	2.14	2.05	1.95
-8.00	2.14	2.04	1.94	1.84	1.74	1.65
-7.00	1.84	1.74	1.64	1.54	1.44	1.35
-6.00	1.54	1.44	1.34	1.24	1.14	1.05
-5.00	1.24	1.14	1.04	0.94	0.84	0.75

SE = spherical equivalent; AZ = ablation zone.

Depending on the magnitude of preoperative spherical equivalent aimed to be corrected and the value of the treatment ablation volume.

Corneal asphericity with an 8-mm corneal diameter was analyzed by linear regression, as shown below.

$${\text{Q}}^{\text{8mm}}{}_{\text{4m}}=\;-0.77\;-\;0.645\;\times {\text{ SE}}_{\text{PRE}}-\;0.273\;\times \text{ AZ }\left(\text{Corneal Wavefront}\right)$$

It was also negatively correlated with the preoperative sphere and AZ. A similar correlation with 8-mm corneal diameter was seen in the Aberration-Free and Corneal Wavefront groups at 4 months postoperatively. Corneal asphericity increased by 0.645 with each diopter of SE correction in the postoperative period, and it reduced postoperatively by 0.273 with each increase of 0.1 mm in the AZ (Table 4).

**Table 4:** Linear regression analysis showing changes in asphericity for 8-mm corneal diameter (Corneal Wavefront).

SE (D)	AZ (mm)					
	7	7.2	7.4	7.6	7.8	8
-9.00	3.12	3.07	3.01	2.96	2.91	2.85
-8.00	2.48	2.42	2.37	2.32	2.26	2.21
-7.00	1.84	1.78	1.72	1.67	1.62	1.56
-6.00	1.19	1.13	1.08	1.03	0.97	0.92
-5.00	0.54	0.49	0.43	0.38	0.33	0.27

SE = spherical equivalent; AZ = ablation zone.

Depending on the magnitude of preoperative spherical equivalent aimed to be corrected and the value of the treatment ablation volume.

## Discussion

The treatment of myopia with LASIK is currently highly popular, because of the rapid positive postoperative visual outcomes and wound healing. In this study, our aim was to compare the visual outcome and predict the postoperative corneal asphericity after femtosecond laser-assisted LASIK employing a six-dimensional excimer laser with the Aberration-Free mode and Corneal Wavefront mode. Use of an excimer laser with a high repetition rate shortens the procedure and reduces the risk for undesired eye movements and postoperative inflammatory reactions or opacities in the interface. [8–10]

In our study, the successful registration rate for SCC was 81% in the Aberration-Free and 90% in the Corneal Wavefront groups. A total of 57% of SCC measurements were within ± 5 degrees, while two eyes in the Aberration-Free group and three eyes in the Corneal Wavefront group showed SCC values >10 degrees in the current study. Smith et al. [11] investigated cyclotorsion in the seated and supine positions. In their study of 30 eyes, they found no significant difference in the axis of astigmatism between patients in the seated and supine position. In 2008, Hyojin analyzed the ocular cyclotorsion according to body position and flap creation before LASIK. [12] We found that cyclotorsion was induced by flap creation. Guirao et al. [13] found that a low degree of cyclotorsion did not cause noticeable effects in the outcome. However, it could have a significant negative impact on the postsurgical outcome with rotations greater than ± 2 degrees.

The refractive outcomes in this study with the aspheric 750 Hz LASIK procedure is predictable, safe, and effective in eyes with myopia or myopic astigmatism. At 6 months, there were 61.6% (Aberration-Free) and 57.2% (Corneal Wavefront) eyes within ± 0.50 D, and 80.8% (Aberration-Free) and 66.7% (Corneal Wavefront) eyes within ± 1.0 D. Our results and those of previously published studies showed that the refractive and visual outcomes of the excimer laser using the AMARIS system are similar. [14]

At 6 months postoperatively, UDVA was 20/20 or better in 77% of eyes in the Aberration-Free groups and 95% of eyes in the Corneal Wavefront groups, while 100% of eyes achieved UDVA of 20/25 or better with both treatment modes. We found that the amount of correction or the amount of pupil offset did not affect the postoperative visual outcomes, similar to the findings of Arba-Mosquera et al. [15] in 2016.

The pupil size changed with a shift in the pupil center. Pupil offset refers to the distance between the corneal vertex and pupil center. In our study, the Aberration-Free mode involves that the pupil center remains the reference for the center of the ablation and the Corneal Wavefront mode involves that the corneal vertex acts as the reference for the optical axis of the ablation. Arba-Mosquera et al. [15] have suggested that for noncoaxial eyes, an aspheric cornea without aberrations (even without astigmatism) will show coma, but those with astigmatism will show trefoil at the pupil area. Under this premise, the corneal aberration of coma and trefoil could be corrected using the Corneal Wavefront mode. Due to a lack of postoperative HOA data, we did not compare the postoperative HOA between two groups. However, there was no significant difference in refractive outcomes at 6 months, between the two different treatment modes.

The induction of HOAs after myopic LASIK is well documented. For example, Alio et al. [14] found that corneal HOA and spherical aberration are statistically significantly induced after myopic LASIK with an AMARIS excimer laser. Dry eye syndrome is one of the most common complications after LASIK. [16] Denoyer et al. [17] investigated dry eye patients and found a significant variation in total corneal HOAs. In 2010, Bottos et al. [18] found that myopic corrections induced changes in the Q value and spherical aberrations and hyperopic corrections reduced changes in the Q value and spherical aberrations. In a previous study by Goyal et al., [19] changes of the Q value at 6-mm corneal diameter were analyzed; there were significantly smaller changes in the aspheric group than in the wavefront-guided group. In our study, the postoperative changes in Q value between the two groups were not statistically significant, but the Corneal Wavefront treatment resulted in a more prolate-shape than did the Aberration-Free treatment mode. We investigated the changes in keratometry readings after myopic correction with the different treatment modes, and found statistically significant flattened in the postoperative steepest keratometry reading (K_2_) in the Corneal Wavefront groups than the Aberration-Free groups.

Linear regression analysis in our study showed that the lowest level of refractive error resulted in fewer changes in the postoperative corneal asphericity for a given AZ. For every 200 μm increase in the AZ intended to treat, the induced changes in postoperative corneal asphericity could be reduced according to the level of myopia requiring correction. A similar result was reported by Vega-Estrada et al. [20] in 2012. They found that corneal asphericity increased by 0.223 in the postoperative period with each diopter of spherical correction; and was reduced by 0.061 in postoperative asphericity with every 0.1 mm increase in the AZ. Savini et al. [21] investigated the influence of corneal asphericity on the refractive outcome of IOL power calculation formulas. They found that a prolate shape can lead to a myopic surprise, while an oblate shape can lead to a hyperopic surprise.

There are some limitations to our study. First, we evaluated only refractive outcomes after myopic correction refractive surgery, such as sphere and cylinder; thus, further analysis is needed for eyes that have undergone hyperopic refractive surgery, and to evaluate the changes of higher-order aberrations after refractive surgery. Second, we obtained postoperative measurements at 6 months. Our research group conducted a study in 2015 in which we evaluated changes in visual and refractive outcomes of moderate myopic eyes over 10 years after refractive surgery, using a VISX S4 excimer laser system. [22] We found that myopic regression after LASIK was corrected with a low residual bed thickness (RBT) preoperatively. In the current study, the mean RBT value was larger than 350 μm in both groups. Miyata et al. [23] reported that the mean RBT value of 388 μm also occurred in the anterior bulging of the cornea after LASIK. Third, we did not investigate which factors make influence in the postoperative refractive outcomes. Frings et al. [24] found that the postoperative manifest SE (MSE) depended on the preoperative difference between cycloplegic SE (CSE) and MSE. In future, we plan to investigate which treatment yields an SE closest to zero MSE postoperatively.

We found that the refractive outcomes and visual acuity were not significantly different between the Aberration-Free mode and Corneal Wavefront mode, but that there was a significant difference in postoperative steepest keratometry readings between the two groups. Linear regression analysis of corneal asphericity is useful for predicting the changes of anterior corneal curvature.
